# Abstract of a Paper on Diagnosis and Surgical Treatment of Empyema of the Maxillary Sinus

**Published:** 1901-09-15

**Authors:** W. H. G. Logan

**Affiliations:** Chicago, Ills.


### ABSTRACT OF A PAPER ON DIAGNOSIS AND SURGICAL TREATMENT OF EMPYEMA OF THE MAXILLARY SINUS.

BY W. H. G. LOGAN, D.D.S., CHICAGO, ILLS.

In the paper the essayist referred to the cause of empyema of the maxillary sinus and said that he did not believe that we had direct penetration of septic matter from the dental alveolar abscesses, as often has been claimed, yet that direct penetration of septic matter does occur at times from this cause can not be denied.

He described the diagnostic symptoms of the disease and advocated the use of the electric light to assist diagnosis.

In regard to the surgical treatment, he believes in a buccal opening on a line with the floor of the antrum.

The objections to operating through the socket of an extracted tooth were enumerated as follows:

1. The frequent needless loss of the tooth.

2. The uncertainty of gaining access to the lowest point of the floor.

3. The liability of missing the sinus entirely and entering the nasal fossa.

4. The removal of diseased hard and soft tissue is accomplished with more difficulty and made improbable in many cases if the opening is not greater in size than the space occupied by one tooth.

5. An ocular examination of the sinus can not be made through the ordinary size opening used for the reception of the tube.

The ideal operation to control empyema of the sinus should have the three following points:

1. The opening made large enough to allow of an occular examination.

2. That the position of this opening be such that it will afford the best natural drainage and not demand extraction of any tooth.

3. The employment of some non-irritating method to maintain the original opening until one can secure a normal condition of the diseased part by the employment of a gutta-percha plug.

In treating, the parts should irrigated daily until cured, employing carbolic acid and nitrate of silver solutions, carrying the fluid with a one or two-ounce syringe, forcing the fluid in until it comes through the nose. If the artificial opening is made very large and low enough for perfect drainage, keeping opening open and irrigated regularly, success will crown your efforts.

This paper was given with stereopticon views and was delivered mostly as a lecture. The essayist showed a number of lantern slides made by Dr. Cryer and himself, and showed the possible results of endeavoring to open into the antrum by the usual methods. The essayist advocated making a considerable opening through the alveolus and maxillary plate by raising the cheek tissue. This gave free access for instrumentation and surgical cleanliness and the remedial applications.

The essayist advocated the use of a gutta-percha plug for closing the orifice during treatment. This plug is made of softened gutta-percha to fit the case and is held in place by a wire which extends down to a tooth which has been banded and fitted with a tube to hold the end of the wire.

DISCUSSION.

Dr. G. V. I. Brown, of Milwaukee: It is surprising that we do not more often have antral pyemia from alveolar abscesses. The reason for this is due to the fact that the abscesses do not disintergrate and penetrate the fibrous mucus membrane which lines the antrum, but lift it up and burrow along on its under side. Do not advocate making large openings into the autrum for all cases of pyemia; most of them can be cured by simple drainage and disinfection. In those cases which require curetting, I believe in making a large opening, and also packing the antrum cavity with sterile gauze. I do not believe the gutta-percha plug can be used without producing undesirable irritation. I sometimes dip the last end of my gauze strip in chloro-percha and this serves to perfectly seal the opening.

Dr. T. L. Gilmer, of Chicago: In opening into the antrum, where curetting or any other operative procedure is required, I make the opening practically as suggested by the essayist, and of such size that the small finger may be introduced to feel the conditions of the walls of the cavity. It is more satisfactory than to explore with a probe. The steel probes are more satisfactory than any other metal, as they reflect sounds better, and the sense of touch is more acurate. Don't open into the antrum with a chisel or anything that will splinter the inner plates of the bone and leave the chips in the cavity. I always pack the antrum with gauze, and do not use a plug at all, and under no circumstances a so-called drainage tube. Have never been troubled by food crowding into the antrum cavity.

Dr. C. P. Pruyn, of Chicago: Approve of large openings and make them always with suitable burs. If the natural opening into the nose should be closed I would make another at the lowest point into the nose so as to secure complete drainage. If there are septi would curette them away. I often use a soft rubber drainage tube. By turning over one end and inserting this end into the antrum it will hold it securely in place and it will also be effective. Use the gauze when indicated, but seldom use the iodoform gauze because of its offensive odor and taste.

Dr. W. A. Cook, of Chicago: Harm is often done by the use of destructive disinfectants in antral work; sterilizing solutions of carbolic acid can not be employed without endangering the vitality of the mucous membrane. Iodoform is not only offensive but inert in most cases. A ten percent solution of chinasol is an efficient germicide and stimulant without destructive irritation. A compound of carbolic acid and sulphuric acid, in proper dilution is much used in Russian hospitals. The acids decompose each other, forming a new acid (a sulphonic phenol), which is a very active disinfectant and without detrimental action.

Dr. W. C. Barrett, of Buffalo, N. Y.: I have no use for antiseptics in antral treatment, except so far as antiseptic results may follow the use of disinfectants and stimulating drugs. For disinfection have had excellent results from using the preparation known as electrozone. It is germicidal and non-irritant. Have also had good results from using nine parts of forty percent alcohol to one part of formaline.

Dr. L. E. Custer, of Dayton, Ohio, thought that often extensive operations in antral work were made when they were not indicated, because a complete diagnosis could not be made. He would recommend the X-ray as the most valuable method of obtaining a correct diagnosis of all hidden diseases.

Dr. Hungerford: Drainage will cure any uninfected antral trouble, and disinfection with drainage and antisepsis will cure any infected antrum. Have found solutions of formaline most efficient of all disinfectants, but must be used well diluted; would also urge the importance of cutting all septi on the floor of the antrum.

Dr. Logan: I never use a flexible rubber tube, it will collapse and not drain. An infected antrum can not be cured by simple drainage; it frequently demands an operation. It is important that a sufficient opening be made at first to enable a correct diagnosis to be made. Use a silver probe to note form and a steel one to detect obstructions.

